# Influence of the Hall Technique on Patient Cooperation: A Retrospective Analysis

**DOI:** 10.3390/jcm14020304

**Published:** 2025-01-07

**Authors:** Ruth M. Santamaría, Amr Gomma, Manasi R. Khole, Christian H. Splieth, Mohammad Alkilzy

**Affiliations:** Department of Paediatric Dentistry, University of Greifswald, 17475 Greifswald, Germany; amrgomma@outlook.com (A.G.); manasi.khole1@uni-greifswald.de (M.R.K.); splieth@uni-greifswald.de (C.H.S.); alkilzym@uni-greifswald.de (M.A.)

**Keywords:** Hall technique, minimally invasive treatment, behavioural change, paediatric dentistry

## Abstract

Child’s cooperation and behaviour in paediatric dentistry are largely determined by the nature of the treatment. Minimally invasive, faster, and more comfortable treatments can lead to greater cooperation and improved behaviour. **Objectives:** To assess the impact of the Hall technique (HT) on children’s behaviour over time across three consecutive treatment sessions through a retrospective analysis. **Methods**: The analysis included children aged 3 to 10 years treated with the HT, with both a pre- and post-Hall technique appointment within a 6-month period. The level of cooperation at each visit was assessed using the Frankl behaviour rating scale (FBRS). The FBRS scores from the three visits: T1 (first treatment session), T2 (Hall technique session), and T3 (follow-up session) were analysed to predict the endpoint (T3–T1) using the Wilcoxon signed-rank test. In addition, an ordinal logistic regression model was used to assess the relationship between variables and behavioural change. **Results**: Of the 90 children included in this study (mean age 5.9 ± 1.56 years; 50 boys, 55.6%), the proportion of patients exhibiting extremely positive/positive behaviour increased steadily over the sessions, from 56.7% at T1 to 76.7% at T2 to 91.1% at T3. The initial behaviour at T1 was the only significant predictor (*p* < 0.0001) of the improvement in behaviour (T3–T1) in the ordinal logistic regression model, which also included variables such as age, sex of the patient, and the treating dentist. **Conclusions**: The Hall technique had a significant positive effect on improving children’s behaviour over the course of treatment.

## 1. Introduction

In paediatric dentistry, the nature of any treatment has a profound effect on a child’s cooperation and overall behaviour during dental procedures. The child’s response to treatment can be influenced by several factors, including previous dental experiences, anxiety levels, and the perceived invasiveness of the procedure. Treatments that are perceived as less invasive, quicker, and more comfortable tend to be associated with better cooperation and more positive behaviour [1]. For instance, minimally invasive techniques, such as the Hall technique (HT), might significantly improve cooperation in children [2]. The HT, named after the Scottish general dental practitioner Dr Norna Hall, was developed in the mid-1990s. It involves placing a preformed metal crown (stainless steel crown) on an asymptomatic carious primary molar without the use of local anaesthetic (LA), caries removal, or tooth preparation [3,4,5,6].

Considering that dental caries is a biofilm-associated disease driven by bacteria, metabolic by-products, and a carbohydrate-derived matrix that produces acids, leading to demineralisation, cavitation, and eventual loss of tooth structure, the HT offers an effective approach. By placing a preformed metal crown over the carious lesion, the biofilm is physically isolated, depriving it of access to carbohydrates and creating an unsustainable environment for bacterial survival, thereby sealing and inactivating the lesion [4]. In addition, the simplicity of the technique, as discussed below, reduces chairside time and increases patient cooperation.

This approach contrasts with traditional ‘drill and fill’ restorative treatments that often require LA and caries removal, which can potentially cause discomfort and anxiety in children, particularly younger ones. Evidence [7] suggests that eliminating the use of LA in paediatric restorative procedures, as seen in techniques such as atraumatic restorative treatment (ART) [8], can significantly reduce discomfort in paediatric patients. Accordingly, the reduced invasiveness of such procedures is associated with better cooperation and more positive treatment experiences for children.

In addition, it may be that the type of dental treatment not only influences the immediate cooperation during the procedure but may influence the child’s cooperation during future dental visits, thus influencing their long-term acceptance of dental care [9]. Positive treatment experiences, especially those that are less invasive, can build trust between the child and the dentist, reducing anxiety and improving cooperation at subsequent appointments. This emphasises the importance of child-friendly treatment approaches in paediatric dentistry [2].

Although there is substantial evidence to support the long-term clinical success and immediate acceptance of the HT, its influence on future patient behaviour and co-operation has not been thoroughly evaluated. This retrospective analysis aims to evaluate children’s behaviour at three treatment sessions: at baseline (before Hall crown placement), during the placement of a Hall crown, and afterwards. By examining these stages, this study aims to determine whether the Hall technique has a lasting effect on children’s behaviour and cooperation during future dental treatment.

## 2. Materials and Methods

### 2.1. Ethical Approval and Study Outline

Ethical approval was obtained from the University Research Ethics Committee (approval number BB 028116). All data were meticulously recorded, managed, and stored securely to ensure accurate reporting, interpretation, and verification. Confidentiality of clinical records was maintained by using unique identification codes for each subject to safeguard privacy.

Using a retrospective design, this study was conducted at the Department of Paediatric Dentistry in a University setting (University of Greifswald, Germany) to assess possible behavioural changes in children treated with the Hall technique (HT).

In general, most of the patients treated in the department are classified as high caries risk, with dmft values of up to 4. The specialised paediatric dentistry department is located in Germany and serves as a regional referral centre. It operates within the framework of the German health care system, which fully covers all necessary dental treatment, including preventive, restorative, and surgical procedures.

### 2.2. Performance of the Hall Technique at the Clinical Setting

Indications: The HT is indicated in proximal, occlusal, or multi-surface, cavitated or non-cavitated carious lesions without symptoms of irreversible pulpitis and have a clear band of dentin between the base of the lesion and pulp on the radiograph; the child presents with limited abilities to cope with another more conventional treatment modality like fillings [4].

Contraindications: these include teeth with pulpal or periapical pathology, severely destructed teeth with no crown structure to retain the crown, where the airway cannot be managed, and fear of aspiration exists [4].

Method of application: After a tooth had been indicated for the HT, preformed stainless steel crowns (©3M ESPE, St. Paul, MN, USA) of different sizes are tried on the indicated tooth until the correct size was found. The correct size is determined by the crown that covers all the cusps and tooth surface and is given a spring back feeling, as described by Innes and colleagues [4]. The tooth is then isolated using cotton rolls and precautions are taken to prevent aspiration, like the placing of a gauze piece. The selected crown is then cemented using a glass ionomer cement (GC Fuji TRIAGE, GC^®^ Corporation, Tokyo, Japan). The seating of the crown is enhanced by the patient by biting on a piece of cotton roll to ensure the proper seating of the crown. The excess cement is then removed, and occlusion is checked. A slightly elevated occlusion is expected as no tooth preparation is performed.

Advantages of the technique: quick, minimally invasive, requires moderate co-operation by patient, and cost-effective as costs are covered by the dental insurance system in Germany.

Limitations: cannot be used in non-co-operative patients, aesthetic limitations due to the silver colour, and cannot be seated in severely tipped teeth where the pre-formed crown cannot be tried or seated due to limited space owing to the tipping of neighbouring teeth.

### 2.3. Outcomes

The primary outcome was the change in behaviour (increase or decrease in cooperation level) following the placement of Hall crowns, as assessed by treating dentists. This was measured using the Frankl behaviour rating scale (FBRS) [10,11]. The FBRS assesses the children’s cooperation by means of four categories, as follows:

- Score 1: Definitely negative behaviour, with refusal of treatment, crying forcefully, fearful, or any other overt evidence of extreme negativism.
- Score 2: Negative behaviour, with reluctance to accept treatment, uncooperative, some evidence of negative attitude but not pronounced, i.e., sullen, withdrawn.
- Score 3: Positive behaviour, cautious acceptance of treatment, displaying willingness to comply with the dentist, at times with reservation but patient follows the dentist’s directions cooperatively.
- Score 4: Definitely positive behaviour, good rapport with the dentist, interested in dental procedures, laughing, and enjoying the procedure.

Prior to this study, a calibration exercise was carried out to ensure that the assessment of the Frankl behaviour scale could be used as a reliable research tool in this retrospective study. Calibration took place in the same university setting. It involved 15 recorded clinical cases from different sessions and countries. Five specialists and twelve postgraduate students participated and independently graded the treatment sessions using the Frankl scale. A highly experienced specialist, previously calibrated in both scales, served as the gold standard. Statistical analysis of the results showed good agreement between the participants, with an intraclass correlation coefficient ICC = 0.715, confirming the reliability of the calibration process.

### 2.4. Inclusion and Exclusion Criteria

**Figure 1:**
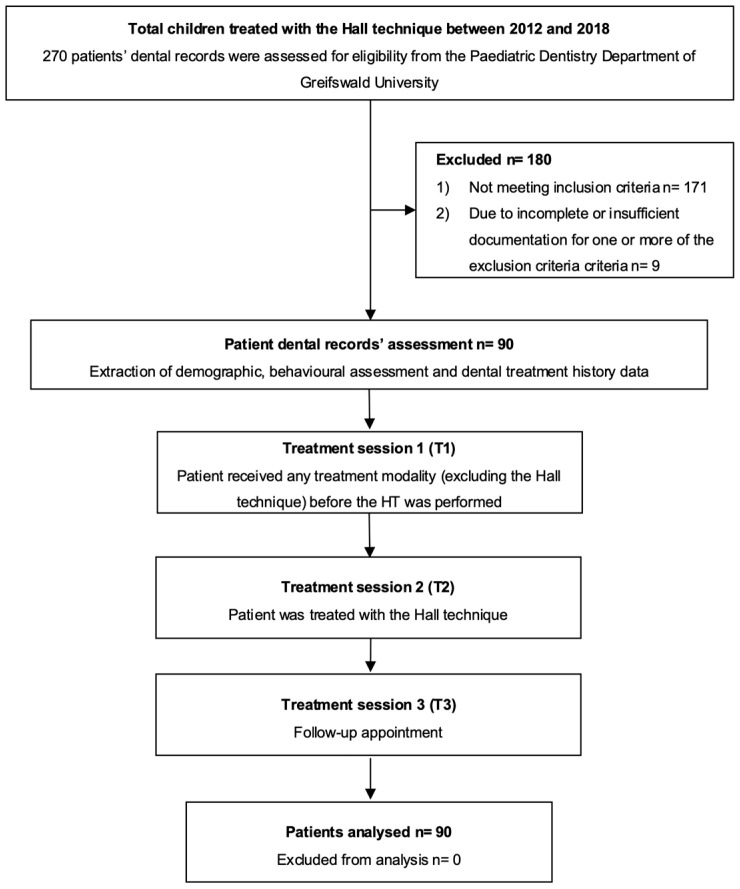
Study Diagram.

Inclusion criteria for this study were children aged 3–10 years with at least one primary molar treated with the HT, with three treatment sessions (T1, T2, and T3), T2 being the appointment when the HT was placed, and a treatment span of 6 months. Exclusion criteria included preformed metal crowns (PMC) placed using the standard technique (injection, caries removal, and tooth preparation) or any modified HT (e.g., removal of proximal contacts, removal of occlusal surfaces, selective caries removal), treatment performed under sedation or under general anaesthesia, and cases without adequate documentation of the patient’s level of cooperation as assessed by the FBRS. Records found to contain inadequate documentation were excluded. The study diagram is shown in Figure 1.

### 2.5. Sample Size Calculation

Sample size was calculated using G*Power 3 software [12]. An F-test was used to compare repeated measures within the same group, with a significance level (α) of 0.05 and power of 80% (1 − β = 0.80). This resulted in an initial sample size of n = 76 patients, ensuring adequate power to detect differences between repeated observations in the cohort, such as changes in treatment cooperation over time.

A previous study in the same setting compared the HT with two other treatment modalities [13]. The HT arm in this study included n = 44 patients. From a clinical perspective, it was considered important to align the sample size of the current study with that of the HT arm of the previous study to support clinical equivalence.

To improve data quality and to address potential issues, such as insufficient documentation affecting data accuracy, a target sample size of 88 children was set. This included a 15% overestimate to ensure that the study retained sufficient power and reliability despite any potential data limitations.

### 2.6. Data Collection and Variables

The patient sample was collected using the electronic record software DAMPSOFT (Version 1.6) in the Department of Paediatric Dentistry at a university hospital. The clinical records of children treated between 2012 and 2018 were reviewed. As many retrospective studies are conducted in this university setting, data are recorded in a standardised format with predesigned text fields to ensure consistency. In addition, all treating dentists, regardless of experience level, receive guidance on data recording practices to improve the quality and reliability of clinical documentation.

Recorded data included demographics (age, sex, health status), baseline clinical findings (d_1–4_mft/s-D_1–4_MFT/S index, clinical diagnosis at tooth level), and treatment provided. Data from three consecutive treatment sessions (T1 [before the HT was performed], T2 [HT appointment] and T3 [follow-up appointment]) were recorded. Additionally, the treating dentist’s qualification (specialist or postgraduate student), and the mode of treatment (chairside) was also recorded. Behavioural ratings were assessed by the dentist at each session using the FBRS to assess any change across the sessions.

### 2.7. Treating Dentist and Treatment Selection

Patients were treated by a team of paediatric dentists (n = 5) and postgraduate paediatric dentistry students (n = 12), all of whom treat children on a regular basis. Each clinician was adequately trained and competent to perform the HT.

At the Paediatric Dentistry Department, treatment selection typically begins after a comprehensive clinical and radiographic assessment of the teeth, and diagnosis is followed by preventive measures, such as plaque disclosure, toothbrushing training for parents and children, fluoridation, and fissure sealants. The patient’s behaviour is a major consideration in guiding the treatment approach and is assessed using the FBRS (see Section 2.1). This behavioural assessment is recorded for each individual session regardless of the type of treatment performed. If the HT is considered as a treatment modality, the technique is then thoroughly explained to the parents and to the child, using child-friendly language to enhance understanding (e.g., describing the crown as a “Princess crown” or an “Ironman crown”).

### 2.8. Statistical Analysis

Data were first entered into a Microsoft Excel 2013 spreadsheet for variable assignment and then transferred to SPSS for statistical analysis (IBM Corp., 2017, IBM SPSS Statistics for Mac, Version 29.0, Armonk, NY, USA). Descriptive statistics were used to summarise patient characteristics. The Wilcoxon signed-rank test for paired observations was used to compare behaviour at different treatment time points (T1 to T3). An ordinal logistic regression model was used to predict outcomes from T1 to T3 [14]. The significance level was set at 0.05.

## 3. Results

### 3.1. Patient Profiles

A total of 270 children were treated with the HT during the study period. Of these, 171 cases (63.3%) were excluded because they did not meet the inclusion criteria. Of the remaining 99 cases, 9 (3.3%) were excluded due to incomplete or insufficient documentation for one or more of the assessment criteria. This resulted in a final sample of 90 patients with a mean age of 5.9 ± 1.56 years. The sample included 50 boys (55.6%) and 40 girls (44.4%). Most patients (n = 62, 68.9%) were referred for treatment.

Of the 90 patients in the sample, three had reported medical conditions that did not influence the treatment decision (diabetes: n = 1, 1.1%; allergy: n = 2, 2.2%). Regarding caries experience, the mean d_3_mft/D_3_MFT was 8.32 ± 3.48/0.31 ± 0.66. When early carious lesions were included, the d_1–4_mft/D_1–4_MFT increased to 11.32 ± 3.71/0.27 ± 0.65. The mean interval between T1 and T3 was 6.2 months, ranging from 2.01 to 6.7 months.

### 3.2. Treatment Characteristics

The treatment characteristics for sessions T1, T2, and T3 are detailed in Table 1. Local anaesthesia was used in a small percentage of cases in T1 (5.6%), with an increase by T3 (14.4%; *p* > 0.05). The majority of procedures in T1 were related to diagnosis and prevention (44.4%), followed by restorative treatments (42.2%). A similar pattern was observed in T3 (*p* > 0.05). However, in T3, approximately 5% more pulp therapy procedures were performed compared to T1. No significant differences were found in the level of specialty among dentists treating in T1, T2, or T3 (*p* > 0.05).

### 3.3. Behavioural Outcomes

The FBRS ratings were recorded over three consecutive sessions (T1, T2, and T3) for each of the 90 patients (Table 2). At T1, 43.3% of the patients exhibited negative or definitely negative behaviour, which later decreased to 23.3% at T2 and further decreased to 8.9% at T3. Conversely, the proportion of patients exhibiting positive or definitely positive behaviour increased steadily over the sessions, from 56.7% at T1 to 76.7% at T2 to 91.1% at T3. Statistically significant differences in behaviour were observed, with a notable increase in patients showing positive or definitely positive behaviour when comparing T1 to T3 (*p* < 0.0001).

**Figure 2:**
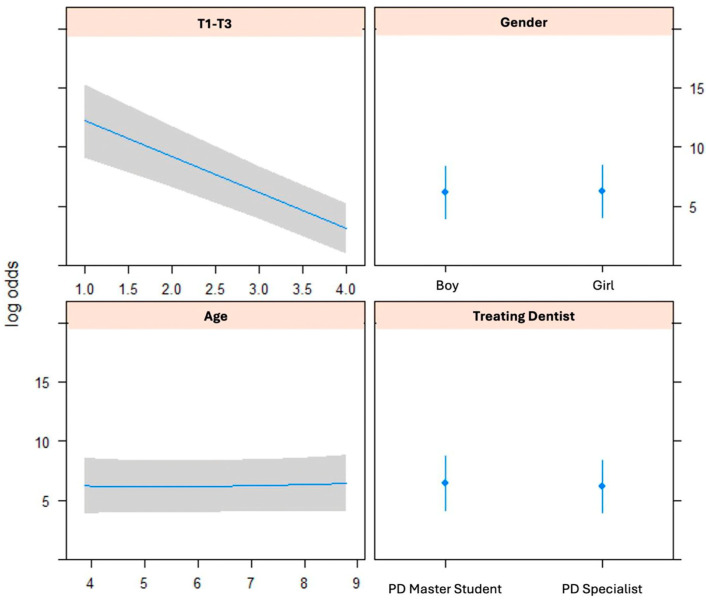
Ordinal logistic regression model for the Frankl’s behaviour rating scale score (definitely negative behaviour), sex, age, and treating dentist.

An ordinal logistic regression model was used to assess whether patient-related factors (age, sex), the type of treatment performed at T1 and T3, or the level of experience of the treating dentist (PD specialist or PD master student) were associated with the outcome. The analysis showed that definitely negative or negative behaviour at T1 (as rated on the FBRS) was the only statistically significant predictor of outcome (F3–F1; *p* < 0.0001). Other factors, including sex (*p* = 0.85), age (*p* = 0.95), and experience of the treating dentist (*p* = 0.67), did not show a significant association with the outcome. See Figure 2.

## 4. Discussion

The results showed a statistically significant improvement in assessed behaviour following the use of the Hall technique (HT), as indicated by the FBRS between the first (T1) and third (T3) sessions (*p* < 0.0001). This improvement was consistent across age and sex and was not influenced by whether the treating dentist was a postgraduate student or a paediatric specialist.

These findings are consistent with previous clinical studies on the acceptability of the HT among children, their caregivers, and dentists [2,15,16,17]. In a randomised clinical trial and some other studies [4,6,15] the ease of use of the HT was discussed, highlighting the advantages of not requiring local anaesthesia, caries removal, and tooth preparation. In this study, general practitioners (GPs) performed the Hall crowns, supporting the notion that a paediatric dental specialist is not required for its application. The current study also found that the level of experience of the dentist (specialist vs. postgraduate students) did not influence behaviour changes.

A related study conducted in Germany [18] compared the acceptability, pain perception, and cooperation levels of children undergoing different treatments [Hall technique (HT), non-restorative caries treatment (NRCT), and conventional restoration (CR)]. This study favoured the HT, with low pain intensity reported in 81% of cases, compared to NRCT (88%) and CR (72%). Unfavourable behaviour was most commonly observed with CR (37%), followed by NRCT (21%) and the HT (13%). In addition, 77% of dentists rated the HT as very easy to perform. Another study conducted in the UK [19] used a pilot trial before setting up a randomised controlled clinical trial to determine the effectiveness of the Hall technique. This study included four general practitioners and four hospital dentists who placed Hall crowns in 45 children. The results showed high levels of satisfaction from both parents and children, suggesting that the technique was well accepted by all involved.

The relationship between age, sex, and fear perception during dental treatment has been analysed in a Korean study [20]. Using a survey-based methodology in 453 school-going adolescents, the authors concluded that girls tend to be more fearful of pain than boys, which may increase their fear of dental treatment. This is concurrent with the results of several other studies, where dental fear and anxiety have been reported to be higher in females than in males [21,22,23]. However, in the present study, the sex of the patient did not show a statistically significant effect on the FBRS ratings when comparing T1 and T3. This suggests that the use of a less invasive treatment approach, specifically the HT, which avoids local anaesthesia may have contributed to the improved behavioural outcomes.

Several studies have found an association between the use of local anaesthesia (LA) and children’s level of cooperation during dental treatment [24,25]. In this study, due to the limited use of LA, statistical analysis did not reveal significant differences in the FBRS scores. However, it is important to note that the primary focus was on overall changes in cooperation rather than acceptance of specific procedures. Previous research has shown that LA is one of the most anxiety-provoking procedures in children’s dental care [25]. It is well known that dental anxiety, cooperation, and behaviour are highly interrelated. Therefore, in cases of low cooperation or limited coping skills, minimally invasive yet effective treatment approaches, such as the HT, should be prioritised, alongside a comprehensive assessment of the child’s character and previous dental experiences [26,27].

The main strength of this study lies in its novel approach to assessing children’s cooperation over three consecutive sessions before and after the HT. Despite its retrospective design, potential biases were minimised among other through rigorous documentation practices at the treatment setting, where behaviour is consistently assessed using the FBRS, enhancing data reliability. Furthermore, postgraduate students performing the HT were well-trained and adhered to a standardised protocol, reducing variability in treatment. Since multiple operators were involved, the results of this study are more pragmatic. However, this study faced limitations due to its strict inclusion criteria, which led to many exclusions. Most exclusions were related to patients missing treatment sessions, ultimately limiting the sample size.

In addition, despite the retrospective nature of this study, a calibration process was carried out to assess whether the involved clinicians could accurately differentiate between the Frankl behaviour categories, giving good results. Considering all the stated limitations, the potential impact of this study’s retrospective design on the outcomes is deemed to be minimised. All operators belonged to the same department and adhered to standardised clinical protocols and a calibration process to ensure consistency of evaluations was performed.

On the other hand, the patient cohort treated at the department exhibits a higher caries experience compared to the general population in Germany. This is due to the fact that the department is a specialised paediatric clinic which receives patients with referrals, and many are severe cases with complex clinical presentations. This context highlights the importance of the HT in the management of children with challenging treatment needs who require specialised behavioural management and treatment approaches to effectively control their caries experience.

Regarding behavioural improvement observed in the present study, it may have resulted from the children’s initial uncertainty about what to expect during the first treatment session (T1), which could have led to higher stress scores. By the second session (T2), increased familiarity with the clinical setting, staff, and procedures might have contributed to reduced stress levels, resulting in better scores. However, given the retrospective nature of the data analysed, it is not possible to objectively measure or isolate this effect within the scope of this study. We speculate that, if T2 had involved any invasive challenging treatment, the positive behaviour observed in T2 would probably not have been maintained and the behavioural outcomes for both T2 and T3 would not have been as satisfactory. This suggests that the non-invasive nature of the HT and its minimally stressful application may have played an important role in promoting better behavioural responses across sessions. To further validate these findings, future research should adopt a prospective study design, allowing for controlled observation of variables and a more detailed analysis of the factors influencing behaviour across treatment sessions. This approach could help to clarify the specific impact of the HT and distinguish it from other contributing factors.

In summary, this study found a significant improvement in children’s cooperation following the use of the HT, highlighting its behavioural benefits in addition to its established clinical benefits [2,4,6,28]. In addition, this study showed no significant differences in cooperation scores between treatments performed by postgraduate students and specialists, reinforcing the simplicity of the HT and its suitability for practitioners with different levels of experience. Neither sex nor age significantly influenced behaviour on the FBRS.

Dental caries is the most widespread oral disease that affects primary and permanent teeth [29,30,31]. In most cases, primary teeth remain untreated due to challenges faced in managing the child while treating the carious lesion [32]. The HT is an effective, time-saving, and easy technique which can be used to treat carious teeth without much hassle; it has proven to be successful for decades. However, only a few dentists can perform this technique, and most patients are referred to specialists. Even among paediatric dentists, the knowledge and training of placing a crown using the HT is limited [33,34]. Further research prospects could assess the knowledge of the HT in general dentists in Germany, enhance the dental curriculum by including the HT in student training, and assess the success and efficacy of this technique in children by general dentists. This in turn could reduce the burden on paediatric dentists, help early caries management in children using minimal invasive techniques, and further reduce the financial burden of treatment on the healthcare system.

## 5. Conclusions

Overall, this study highlights the importance of considering both the type of treatment and the psychological aspects of paediatric patients and suggests that less invasive techniques, such as the HT, may positively influence behaviour and cooperation, regardless of the gender differences traditionally noted in anxiety perception. Such positive behavioural improvements could help the healthcare system by enabling the treatment of most children chairside without the need of additional means, like general anaesthesia, which not only pose a death risk to the patients but prove to be a financial burden on the healthcare system.

## Figures and Tables

**Table 1:** Characteristics of the treatments carried out according to the session.

Treatment Session/Variables	Treatment Session 1 (T1)					Treatment Session 2 (T2)		Treatment Session 3 (T3)			
Dentist Treating	PD Specialists n (%)		PD Postgraduate Students n (%)			PD Specialists n (%)	PD Post-graduate Students n (%)	PD Specialists n (%)		PD Postgraduate Students n (%)	
	21 (23.3%)		69 (76.7%)			26 (28.9%)	64 (71.1%)	25 (27.8%)		65 (72.2%)	
Local Anaesthesia Used	5 (5.6%)					0 (0%)		13 (14.4%)			
Treatment Performed	Diagnosis/prevention n (%)	Restorative procedure n (%)	Extraction n (%)	Pulp Therapy n (%)	Other n (%)	HT n (%)		Diagnosis/prevention n (%)	Restorative procedure n (%)	Extraction n (%)	Pulp Therapy n (%)
	40 (44.4%)	38 (42.2%)	5 (5.6%)	0	7 (7.8%)	90		34 (37.7%)	46 (51.1%)	5 (5.6%)	5 (5.6%)

PD = Paediatric dentistry; HT = Hall technique.

**Table 2:** Distribution of patients across the three treatment sessions categorised by the four levels of the Frankl’s behaviour rating scale.

Frankl’s Behaviour Rating Scale	T1	T2	T3	
	n (%)	n (%)	n (%)	*p*-Values
Definitely negative	18 (20%)	3 (3.3%)	1 (1.1%)	*p* < 0.0001
Negative	21 (23.3%)	18 (20%)	7 (7.8%)	
Positive	23 (25.6%)	43 (47.8%)	23 (25.6%)	
Definitely positive	28 (31.1%)	26 (28.9%)	59 (65.5%)	*p* < 0.0001
Total	90 (100%)	90 (100%)	90 (100%)	

Wilcoxon signed-rank test for paired observations. T1 = Treatment session 1; T2 = Treatment session 2; T3 = Treatment session 3.

## Data Availability

The data are contained within this article. Further inquiries can be directed to the corresponding author.
